# Differential expression of Dusp1 and immediate early response genes in the hippocampus of rats, subjected to forced swim test

**DOI:** 10.1038/s41598-023-36611-5

**Published:** 2023-06-20

**Authors:** Ivan Vlasov, Elena Filatova, Petr Slominsky, Maria Shadrina

**Affiliations:** Institute of Molecular Genetics of National Research Centre, Kurchatov Institute,

**Keywords:** Gene expression, Genetics research, Experimental models of disease

## Abstract

The forced swim test (FST) is widely used to screen for potential antidepressant drugs and treatments. Despite this, the nature of stillness during FST and whether it resembles “depressive-like behavior” are widely debated issues. Furthermore, despite being widely used as a behavioral assay, the effects of the FST on the brain transcriptome are rarely investigated. Therefore, in this study we have investigated changes in the transcriptome of the rat hippocampus 20 min and 24 h after FST exposure. RNA-Seq is performed on the hippocampus tissues of rats 20 min and 24 h after an FST. Differentially expressed genes (DEGs) were identified using limma and used to construct gene interaction networks. Fourteen differentially expressed genes (DEGs) were identified only in the 20-m group. No DEGs were identified 24 h after the FST. These genes were used for Gene Ontology term enrichment and gene-network construction. Based on the constructed gene-interaction networks, we identified a group of DEGs (*Dusp1*, *Fos*, *Klf2*, *Ccn1*, and *Zfp36*) that appeared significant based on multiple methods of downstream analysis. *Dusp1* appears especially important, as its role in the pathogenesis of depression has been demonstrated both in various animal models of depression and in patients with depressive disorders.

## Introduction

The forced swim test (FST) is a widely used assay in the fields of neuropharmacology and study of depression. All in all, more than 6000 papers using the FST have been published. The FST was presented by Porsolt et al. as a model created to satisfy two main requirements: they “resemble depressive illness and are selectively sensitive to clinically effective antidepressant treatments”^1^. The sensitivity of the FST to contemporary antidepressant treatments was demonstrated in an original paper^1^. However, the validity of the FST as a model of depressive behavior is now contested. On the one hand, the interpretation of animal stillness during an FST as “depression-like behavior” is most common. It is accepted by 70% of researchers publishing studies using the FST^2^ On the other hand, according to a number of authors, the FST is lacking in the face and construct validity^2^. The FST’s predictive validity is also limited – in the case of some widely used clinical treatments (barbiturates and benzo-diazepines), it has been prone to delivering false-positive results^3^. In addition, significant differences in pharmacodynamics have been observed in human subjects compared with rodents used in an FST^4^. Some authors believe that interpreting the FST as a model of acute stress and adaptive reactions to acute stress would be a more valid approach^2,5^.

However, the importance of stress in the pathogenesis of depression should not be underestimated. The theory of the stress-induced pathogenesis of depression is one of the main theories of depression. In this theory, it is supposed that the hyperactivity of the hypothalamic–pituitary–adrenal axis under the influence of acute stress is a major factor in the pathogenesis of depression. The fact that stressful events are among the main factors that trigger the first manifestations of depression speaks in favor of this theory^6–8^. Additionally, cortisol levels in blood plasma and cerebrospinal fluid, as well as corticotropin levels in blood plasma, are elevated in patients with depressive disorders^9^. Additionally, a reduction in central corticosteroid receptors has been observed in patients with depression^10^.

In view of these data, the use of the FST as a stressor to investigate the effects of acute stress on brain processes and their possible relationship with the pathogenesis of depression appears to be an interesting line of study. Many recent studies employing the FST have examined the effects of antidepressants^11–13^, other drugs (e.g. the hypotensive drug Telmisartan)^14^ other compounds (e.g. milk lactoferrin)^15^, mutations^16^ and physiological factors^17^, by comparing low immobility with high immobility in subgroups. Only a few studies have examined the direct effect of acute stress from the FST on transcription profiles by comparing a group subjected to the FST with a group not subjected to the FST. Therefore, we studied transcriptional profiles in the brains of rats exposed to the FST and divided into groups with immediate (20 min after the test) and delayed (24 h after the test) responses to acute stress.

## Results

A transcriptomics analysis was performed on hippocampal tissues dissected from 18 rats, including six control rats that were not subjected to the FST, six rats decapitated in 20 min (hereinafter 20-m group) after the test, and six rats decapitated in 24 h (hereinafter 24-h group) after the test. Fourteen DEGs were identified in the 20-m group: two were downregulated and 12 were upregulated (see Supplementary File 1 for the full list). No DEGs were identified in the 24-h group. A Gene Ontology (GO) term enrichment analysis was performed using the DEGs from the 20-m group. The following terms were identified as significantly enriched (Table 1).

**Table 1:** Significantly enriched GO Biological process terms.

Term GO ID	GO term	P value (FDR corrected)	Total genes linked to the term	Total DEGs linked to the term (%)	Names of linked DEGs
GO:0071409	Cellular response to cycloheximide	5.3*10–6	4	2 (50%)	[Klf2, Klf4]
GO:0071499	Cellular response to laminar fluid shear stress	8.8*10–6	5	2 (40%)	[Klf2, Klf4]
GO:0060213	Positive regulation of nuclear-transcribed mRNA poly(A) tail shortening	4.8*10–5	11	2 (18, 18%)	[Btg2, Zfp36]
GO:0036003	Positive regulation of transcription from RNA polymerase II promoter in response to stress	9.2*10–5	15	2 (13, 33%)	[Atf3, Klf2]
GO:0006094	Gluconeogenesis	8.1*10–4	55	2 (4, 55%)	[Atf3, Sik1]

DEGs for enrichment are detected in hippocampus of rats from FST 20 m group.

**Figure 1:**
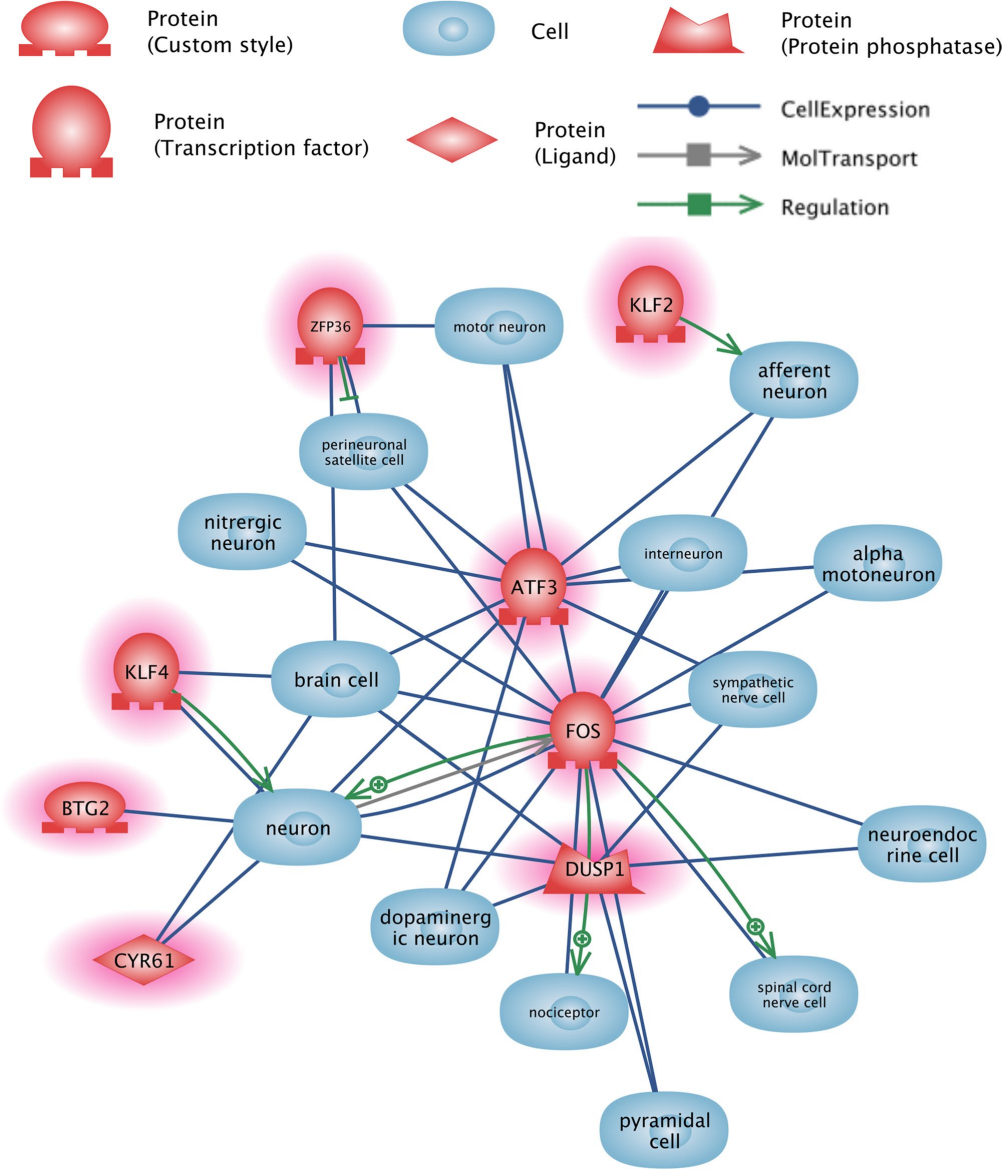
DEGs detected in hippocampus of rats from FST 20 m group and their interactions with various neuronal cells.

We created a network of the interaction of DEGs with various neuronal cells using Pathway Studio (Fig. 1). To construct this network, we determined the relationship between all DEGs and cells found using the keyword “neuron” in Pathway Studio. This network contained eight genes: Cyr61/Ccn1, Dusp1, Klf2, Klf4, Atf3, Zfp36, Btg2, and Fos.

**Figure 2:**
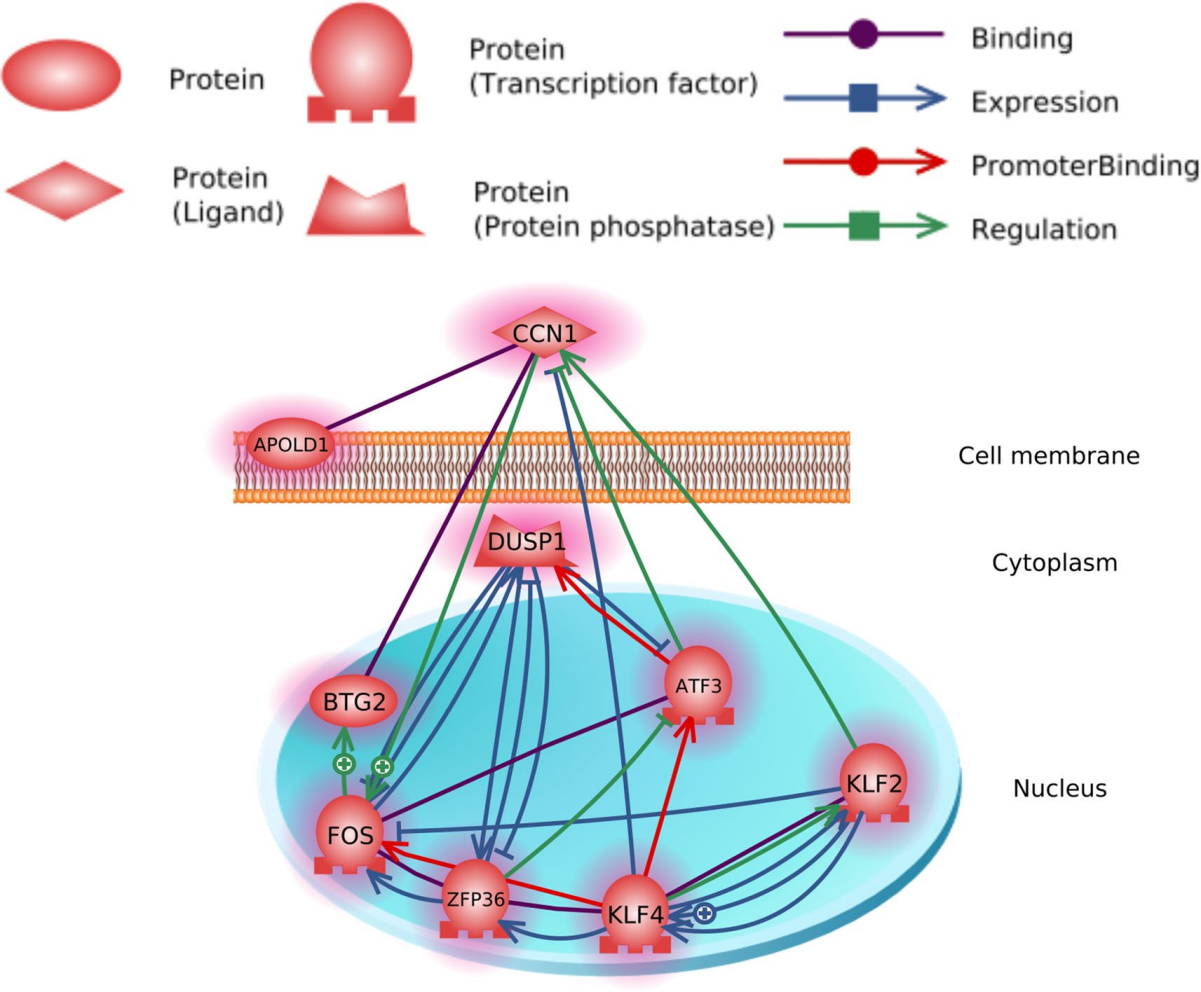
Interactions of DEGs detected in hippocampus of rats from FST 20 m group.

**Figure 3:**
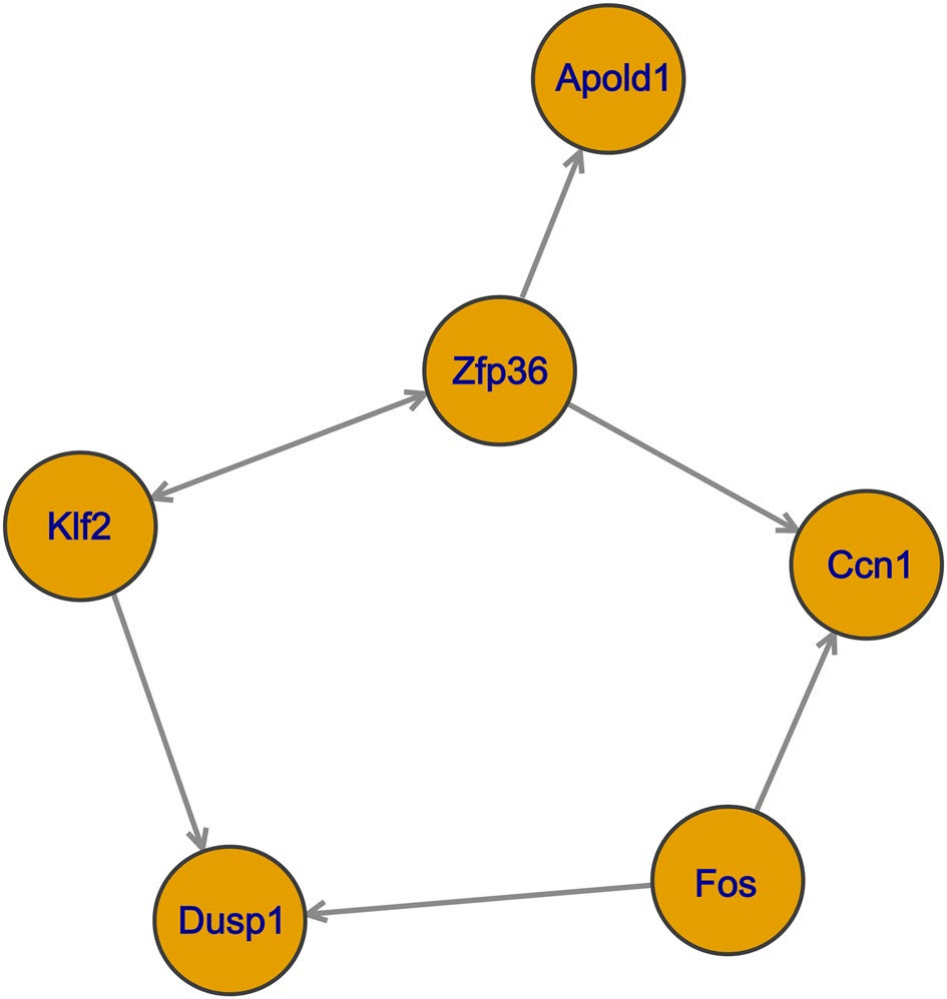
Expression based interaction network of genes in hippocampus based on expression data from FST 20 m, FST 24 h and control group.

Using the Pathway Studio software, we examined the DEGs for any known interactions and identified a group of interacting DEGs (Fig. 2). For this purpose, all known interactions between the DEGs were identified, with all DEGs not interacting with any other DEG being removed. In comparing Figs. 1 and 2, it became evident that eight genes–i.e. *Dusp1*, *Zfp36*, *Klf2*, *Klf4*, *Ccn1/Cyr61*, *Btg2*, and *Fos*––were common for the two networks. Using the “rtn” package in R, we analyzed the coexpression patterns to identify interacting genes based on empirical evidence (Fig. 3). Using expression data (pseudocounts per transcript in each observation), we identified a network of interacting genes that linked transcription factors to their potential targets. This network consisted of the *Apold1*, *Zfp36*, *Ccn1/Cyr61*, *Fos*, *Klf2*, and *Dusp1* genes; all of them were also differentially expressed.

We identified a group of genes (*Dusp1*, *Fos*, *Klf2*, *Ccn1* and *Zfp36*) that appeared significant based on multiple methods of downstream analysis, and examined their possible differential expression in the hippocampus and prefrontal cortex (PFC) using reverse transcription polymerase chain reaction (RT–PCR) (Table 2). Only one gene (Zfp36) was not significantly up-regulated in hippocampus tissues of the 20-m group, although it is worth noting that the direction of the differential expression at the significance level was in accordance with the ribonucleic acid (RNA) sequence data.

**Table 2:** Mean (RNA-seq) and median (RT-PCR) FC of the most interesting DEGs based on RT-PCR and RNA-seq.

Genes	Hippocampus				Prefrontal cortex	
	20 min		24 h		20 min	24 h
	RT-PCR	RNA-seq	RT-PCR	RNA-seq	RT-PCR	RT-PCR
Dusp1	**2.25 (2.18–2.55)**	**3.33**	1 (0.9–1.26)	1.12	**2.71 (2.32–2.85)**	**2.36 (2.13–2.82)**
Fos	**4.83 (2.3–7.05)**	**2.40**	1.58 (1.46–1.97)	1.03	2.62 (1.58–3.76)	**2.94 (2.64–7.43)**
Klf2	**1.9 (1.73–3.11)**	**2.10**	1.5 (1.17–1.83)	1.09	**2.8 (2.46–3.33)**	1.87 (1.53–2.26)
Zfp36	1.47 (1.02–3.82)	**2.17**	1.05 (0.94–1.31)	1.28	**2.26 (1.97–2.65)**	0.87 (0.72–0.94)
Ccn1	**8.05 (4.15–14)**	**10.09**	0.84 (0.65–0.98)	1.04	**11.24 (8.59–15.71)**	1.9 (1.56–2.77)

Significantly DE values are in bold (P value < 0.05) (Kruskal–Wallis test in case of RT-PCR and Moderated t test in case of RNA-seq). FDR multiple test correction is applied in both cases.

## Discussion

In this study, a transcriptomics analysis of the hippocampus tissues of rats subjected to the FST was conducted. The FST is a commonly used model, which is mostly employed in drug screening for investigating potential antidepressant application (according to (Molendijk, de Kloet^5^), no less than 4300 papers in 2015). However, studies of the direct effect of the FST on brain transcriptomes are exceedingly rare.

We conducted a search for similar experiments in the literature data using search queries “stress mouse RNA-Seq” and “stress rat RNA sequence” in PubMed. The results were 757 and 153, respectively. To identify the most relevant papers, we filtered out papers in languages other than English (8); papers with model animals other than rats and mice (63); papers with conditions other than stress or depression, such as cancer, infectious diseases, etc. (497); papers using other tissues than central nervous system, such as bones, lungs, etc. (182); papers investigating other forms of stress, such as endoplasmic reticulum stress or fluid shear stress (15); papers not intended to investigate differential expression (36); papers investigating rats or mice that have been genetically modified, carry significant mutations or deletions, are old, or have been bred for neurological issues (such as being prone to seizures) rats (5); and, finally, papers in which transcriptomic profiling of stressed and nonstressed mice and rats was not conducted (94). Using this approach, ten papers with the most similar conditions and approaches have been selected. Only one paper out of the ten studied differential expression in the brain of mice subjected to FST and not subjected to the FST using RNA-Seq. Nine of the ten papers have studied other models of stress in rodents, such as uncontrollable chronic stress, social defeat, and acute and chronic immobilization.

Zhang et al. in their 2019 paper^18^, conducted a transcriptome analysis in the hippocampus of mice that had been subjected to three different tests–the tail suspension test, FST, and elevated labyrinth test. The authors identified more than 200 DEGs in each test group. The best fit to our own data was the FST test group. In Zhang et al. paper, tissues were extracted from mice 24 h after FST, the same as for our 24-h group. However, unlike our data, 224 DEGs were identified in that test group. Biological differences between rats and mice, methodological differences such as use of pulled mRNA (in (Zhang et al.^18^) vs individual samples in our study), and use of different sets of bioinformatic tools could explain these differences. Unfortunately, lack of access to primary data makes further comparison of results impossible.

A 2021 study (Mifsud et al.^19^, mostly focused on MR/GR regulation and binding, investigated, among other things, the effects of forced swimming stress on gene expression in rat hippocampus. Multiple methodological differences complicate the direct comparison. We consider the following differences to be the most impactful (in (Mifsud et al.^19^) and our work, respectively): the use of isoflurane anesthesia versus no anesthesia, the use of response measurement between several timelines after the FST and two separate baseline groups versus pairwise comparison against a single baseline group, the use of riboRNA depletion versus poly-A enrichment, and the use of a single occurrence of a forced swimming stressor versus use of a pretest swimming stressor before the actual test. Nevertheless, despite these differences, we have identified common genes between their responsive to stress genes and our differentially expressed genes: *Sik1*, *Btg2*, *Atf3*, *Apold1*, *Fos*, *Ccn/Cyr61*, *Klf4*, and *Zfp36*. In both studies, these genes are expressed at a higher level in stressed versus nonstressed rats.

A similar situation can be observed in (von Ziegler et al.^20^). In this work, multiomic profiling was performed on the hippocampi of mice, subjected to a forced swimming stressor at several time points. Despite numerous methodological differences (the biological difference between mice and rats probably being the most important one), the genes *Fos*, *Dusp1*, *Sik1*, *Btg2*, *Atf3*, *Apold1*, *Cyr61*, *Klf2*, *Klf4*, and *Ier2* and their mouse orthologs, respectively, are up-regulated shortly after exposure to the forced swim test in both our own (20 min after the end of the FST) transcriptomic profiling and that of von Ziegler et al. (45 min after the beginning). It is worth noting that, at the latest time point (4 h) in the Ziegler study, the expression of these genes returns to the baseline level, which is consistent with our own measurements at 24 h after the FST.

We have found two studies using microarrays to find changes in brain transcriptome after the FST, including changes in the hippocampus. In this study, 1298 DEGs were detected, which were almost two orders of magnitude higher than in our data^21^ However, these DEGs were expressed insignificantly differentially, which makes comparison with our data incorrect, as we reported only genes that were significantly deferentially expressed after FDR correction.

Similarly, no mention of multiple hypothesis correction can be found in the article (Yamamoto et al.^22^), although in this case a different choice of examined tissue (from the cerebellum and PFC versus hippocampus) has further complicated the comparison. No common DEGs could be found between our results and those reported in this paper.

In many studies, the FST was considered a model of depression and passive animal behavior observed during the test was considered “depressive.” Several articles have criticized the use of FST as a model of depression. An argument used in these works was that passive behavior recorded during the test was adaptive, as the animal recognizes that escape attempts are futile^5,23^. These hypotheses about animal response are consistent with our results; e.g. a transcriptomics analysis of hippocampus tissues from the brains of rats subjected to the FST detected 14 DEGs 20 min and no DEGs 24 h after the test. These results would be expected if the working hypothesis was that animals are not in a depressive state, but simply learn a new adaptive behavior. In contrast, the detection of DEGs 20 min after the test suggests the possibility of using the FST model for the examination of the impact of acute stress on the brain transcriptome.

The amygdala, prefrontal cortex (PFC), and hippocampus appear to be the most promising regions in the brain for the investigation of stress effects on the transcriptome level. The presence of glucocorticoid and mineralocorticoid receptors (GRs, MRs) in these three brain regions is well documented^24^. Neurons in all these regions are affected by remodeling due to stress effects^24^. Therefore, we have decided to use the hippocampus for transcriptome profiling and then confirm the differential expression of several DEGs revealed by transcriptome profiling in both the PFC and hippocampus.

The inhibitory effects of stress and cortisol (CORT) on hippocampus-based memory are widely known^25,26^. Long-term potentiation was also shown to be inhibited under the effects of stress^27,28^. It is worth noting that the effects of cortisol on potentiation and memory are dose-dependent and biphasic: small doses improve memory based on the hippocampus and long-term potentiation, while the effect of high doses is the opposite^29,30^. Furthermore, prolonged stress exposure led to decreased hippocampus volume^31^.

Similarly to hippocampus-based memory, PFC-based working memory is also affected by cortisol and stress in a dose-dependent biphasic manner. Acute stress also improves working memory^32^, and chronic stress inhibits it^33^. Stress exposure causes changes in the morphology of pyramid neurons in the PFC^34^. According to fMRI imaging, individuals under a high level of stress exhibited reduced functional connectivity in brain circuitry, including the PFC^35^.

We performed GO term enrichment for the 14 detected DEGs (Table 1). Due to the low overall number of DEGs detected, even significantly enriched terms had no more than two associated DEGs. Therefore, it was difficult to evaluate the relevance of these terms, especially “gluconeogenesis,” for which two genes represented only 4.6% of all the genes associated with the term. However, the terms GO:0036003 and GO:0071499 are tangentially relevant to stress and may deserve attention. It is also worth noting that the term GO:0071499 (cellular response to laminar fluid shear stress) can be linked to acute stress through common effector mechanisms, i.e. mitogen-activated protein kinases (MAPKs)^36^, the functions of which are highly affected by *Dusp1*, one of the detected DEGs. Additionally, for all 14 detected DEGs, we built two interaction networks including relation to neuronal cells (Fig. 1) and known interactions from the literature (Fig. 2). Moreover, we built a coexpression-based network of interacting genes using transcriptomic profiles (Fig. 3). The genes common between these three networks were chosen for further examination: *Dusp1*, *Fos*, *Klf2*, *Ccn1*, and *Zfp36*.

*Dusp1* is a dual-specificity phosphatase that belongs to a family of phosphatases that inactivate MAPKs, a family of key regulators of cellular processes and the immune response^37^. Based on the literature data, *Dusp1* overexpression appears to be connected to both acute and chronic stress. Meta-analysis of microarray transcriptomic data has showed that *Dusp1* expression was consistently regulated by glucocorticoids^38^. Even in the absence of glucocorticoids, restraint stress by itself leads to the induction of *Dusp1* expression in the hypothalamic paraventricular nucleus and anterior pituitary of adrenalectomized mice^39^. Treatment with dexmedetomidine attenuates both stress-induced liver injury and *Dusp1* overepxression in mice, subjected to acute stress^40^. A similar result, i.e. alleviation of the pathologic phenotype and overexpression of *Dusp1*, has also been shown in a mouse model of learnt helplessness after deep transcranial magnetic stimulation^41^. *Dusp1* is also overexpressed in models of depression caused by chronic pain, and mild, unpredictable, and periodic photostimulation. Intake of a Dusp1 antagonist and local silencing or knockout of Dusp1 alleviated depressive behavior in these models^42^. *Dusp1* was found to be overexpressed in the hippocampus^41,43–45^ and ventrolateral orbital cortex^46^ of rats and mice, subjected to chronic stress. *Dusp1* was also overexpressed in gastric tissues of rats with stress-induced gastric ulcer^47^.

*DUSP1* overexpression also appears to be involved in depression. An increased amount of DUSP1 in blood plasma is associated with depression in women^48^. *DUSP1* is overexpressed in postmortem tissues of the subregions of the hippocampus in patients with major depressive disorder (MDD)^45^. *DUSP1* expression in the blood of patients with MDD is reduced after transcranial magnetic stimulation, a treatment that also alleviates symptoms of depression^49^.

It can be concluded that overexpression of *DUSP1* is central to the physiology of stress. Overexpression of *Dusp1,* linked to a depressive-like phenotype, is a common feature of various rodent chronic-stress-based models of depression. Our data demonstrated there to be *Dusp1* overexpression in the hippocampus and PFC of rats subjected to the FST.

*Fos* is a gene that encodes the immediate early response transcription factor c-Fos. It’s expression is induced as part of an activity-regulated gene expression program in response to various stimuli. It is widely regarded as a marker of neuronal activity^50,51^. Therefore, *Fos* induction as part of the stress response is not a specific reaction to stress, which is well documented in the literature. A comprehensive review of all studies on *Fos* induction in response to stress is beyond the scope of this paper. Therefore, we decided to focus on studies that illustrate the specificity of the connection between *Fos* and stress.

Stress induces *Fos* expression in several regions of the brain involved in stress reaction and regulation of the stress—the hippocampus^52–56^; hypothalamus, amygdala, and nucleus accumbens^52–54,56–60^; HPA-axis^58^; and PFC^53,57,61^. Ingestion of antidepressants and anxiolytics reduces the induction of *Fos* expression. c-Fos is expressed in response to electroshock tests in rodents, and during presentation of the environments in which these tests occurred. Introduction of diazepam reduces induction of this expression^62^. Clozapine restores normal social interactions in rats subjected to acute restraint stress and reduces *Fos* expression in several parts of the brain^53^. Short-term fluoxetine treatment reduces *Fos* expression in the lymbic system of rats subjected to acute forced restraint stress^59^. It is worth noting that the dynamics and localization of Fos induction may be sex-dependent^55,63^. Therefore, common experimental designs using exclusively male rodents (as in our study) may be flawed or limited.

Overexpression of Fos in the hippocampus of rats subjected to the FST may evidence the commonality of the molecular mechanisms of various acute stress models. It is also worth noting that forced swimming and restraint stress induce similar levels of GR/MR binding to the glucocorticoid-responsive elements of specific stress related genes in hippocampus^64^. This commonality makes the FST potentially very useful for research into the molecular mechanisms of stress, considering how easy to conduct and standardize it is.

Klf2, another DEG in our data, is a gene from the Krüppel-like factor family, i.e. DNA-binding transcription factors with zinc finger motifs. The genes of this family participate in the facilitation of different cellular functions. Klf2 plays a role in such cellular processes as inflammation and functioning of the immune system and participates in the differentiation of various cell types^65^.

It has been shown previously that multihit early life adversity causes differential expression of *Klf2* in mice. Interestingly, there is another gene that is regulated in a sex-dependent manner, and the direction of its significant differential expression is opposite in males and females^66^. Our data indicate that there may be a connection of overexpression of this gene with acute stress.

Cysteine-rich angiogenic inducer 61 (*Cyr61/Ccn1*) belongs to the CCN family of genes, a group of regulatory genes connected by a common multimodular organization^67^. Ccn1 was first discovered as an immediate early response gene induced by growth factors^68^. It participates in a multitude of cellular processes, such as angiogenesis, cellular adhesion, proliferation, and migration. Various stressors, such as hypoxia, mechanical stretching, and UV light, induce the expression of this gene. *Cyr61* is up-regulated in various animal models, such as asthma, lung fibrosis, hyperoxia, and ventilator-associated lung injury^69^.

Dieckmann et al. published a study, in which they investigated the potential effects of early childhood adversity on adults. In this study, they compared the transcriptomic profiles of blood monocytes from adults who suffered from early childhood adversity and matched controls 3 h after exposure to a stressful event. In that study, *CYR61* was found among significantly overexpressed genes^70^*.*

Cyr61 expression has previously been shown to increase significantly increased in the neocortex of mice by both restraint stress and intake of FG7142, which is a partial inverse agonist of benzodiazepine receptors that mimics the physiological and neurochemical changes induced by stressful stimuli^71^. In both cases, the mice were sacrificed an hour after exposure. In the same study, *Btg2* and *Fos* were also overexpressed after both stress exposure and FG7142. These two genes were also overexpressed in our study. This could be understood as a demonstration of the commonality of molecular mechanisms involved in stress, caused by FST and other classic stress models, such as restrain stress. This underlines the role of these three genes in the early response to stress.

The last DEG of particular interest encodes Zfp36, also known as tristetraprolin, which is a transcription factor with a zinc finger motif. This protein is an RNA-binding protein that plays a role in various cellular processes, namely differentiation, proliferation, carcinogenesis, and many others^72^. A study performed using the orbitofrontal cortex brain tissues of suicide victims showed the overexpression of *ZFP36*^73^. However, further studies were unable to confirm that there is a connection between a single-nucleotide polymorphism (SNP) of this gene and suicidal behavior^74^. We have detected an overexpression of this gene in the brains of rats subjected to the FST, which implies a connection between changes in the expression of this gene and stress. The potential link between stress, *ZFP36* overexpression, and suicidal behavior may be an important topic for further investigation.

Intergenic interactions among DEGs are also of interest, in particular, the effect of differential-expression transcription factors on the overexpression of other genes. As is shown in Fig. 2, according to data in the literature, many of these genes have a negative impact on the expression of their target genes; however, in our results, all of these genes were overexpressed simultaneously. Using the reconstruction of transcriptional regulatory network inference (RTNI) method, we attempted to recreate the networks of mutual effects on the expression of our DEGs (Fig. 3). In the RTNI analysis, we used all the expression data for all genes. Pathway Studio only worked with DEGs that were previously annotated. Our results appear to confirm a close interaction among *Dusp1*, *Fos*, *Klf2*, *Ccn1*, and *Zfp36*. Preexisting literature data based on the pattern of coexpression support the assumption of the importance of this group of genes for developing acute stress at the molecular level.

## Materials and methods

### FST model

This study was conducted with male Sprague Dawley (SD) rats. Rats weighing 140–210 g at the beginning of the experiment were divided into three groups: a control group, a group that would be decapitated 24 h after the FST, and a group that would be decapitated 20 min after the FST (*n* = 8 animals each). The experimental work was carried out in accordance with the *Guide for the Care and Use of Laboratory Animals*^75^ and was approved by the Ethics Committee of the Institute of Molecular Genetics of the Russian Academy of Sciences. On the first day of the experiment, animals from the first and second experimental groups were placed for 15 min in a cylinder with a diameter of 20 cm and a height of 45 cm that was filled with water at a temperature of 24–25 °C water up to the 30-cm mark (pretest). On the second day, the animals were placed in the cylinder for 5 min (test). Twenty minutes after the second session, the animals in the first experimental group were decapitated. The animals of the second group were decapitated 24 h after the test.

The study was conducted and reported in accordance with the ARRIVE Essential 10 guidelines ^76^.

#### Randomization and blinding

Animals were weighed, and their sucrose preference was measured immediately after their arrival to IMG vivarium and 2 weeks before the FST. These measurement points are called the “first time point” and “second time point” (_1 and _2 in Supplementary File 3).

The animals were randomly divided into groups at second time point using the following procedure:

1. Animals were randomly divided into groups (sample() command in R programming language)
2. The Kruskall–Wallis one-way ANOVA test was used to ensure that groups are not significantly different in sucrose preference and food consumption. If there was a significant difference, the procedure (kruskall.test() command in the R programming language) was restarted.

The same procedure was used to select animals for sequencing, albeit with selection of subgroups of full groups.

Blinding was present during the handling of the animals prior to treatment. Blinding during the carrying out of the experiment, data analysis, and outcome assessment are not applicable to our experiment design. Full data describing the groups can be found in Supplementary File 3.

### RNA isolation

The hippocampus and prefrontal cortex were dissected from the whole brain. The dissection took no more than 10 min per animal. Samples were flash frozen, homogenized a using rotor homogenizer, and then soaked in RNA-later buffer (Thermo Fisher Scientific).

Total RNA was isolated from a 10-mg sample of tissue from the frontal cortex (F) and hippocampus (H) of the brains of rats. Total RNA isolation was performed from the rat brains for expression analysis of individual genes for each individual animal. Total RNA isolation was conducted using a Direct-Zol RNA Mini-Prep Kit (Zymo Research) according to the manufacturer's recommendations. The concentration of isolated total RNA was measured using a Quant-iT RNA BR Assay Kit and a Qubit fluorimeter (Invitrogen). RNA quality was monitored using an Experion automated electrophoresis system (Bio-Rad Laboratories). The RNA quality index, measured using BioAnalyzer (Agilent), was higher than 8.5 in all samples.

Whole-transcriptome analysis was performed using samples of tissue isolated from hippocampus of the rats brain. For this purpose, 5 mg of brain tissue were taken from each of the 8 animals in each group (the control group, the group decapitated 20 min after the FST, and the group decapitated 24 h after the FST).

### RNA sequencing

A polyA fraction of RNA was extracted from total RNA for RNA-seq. Sequencing libraries were prepared using a NEBNext® mRNA Library Perp Reagent Set (NEB, United States). Sequencing was performed using HiSeq1500 (Illumina, United States). Read counts per sample can be found in Supplementary File 4. RNAs of six (FST 24 h, control) or seven (FST 20 min) animals from each group were sequenced.

### RT-qPCR

Gene-expression analysis was conducted using a reverse-transcription (RT) reaction and real-time quantitative PCR (qPCR) with TaqMan probes. Single-stranded DNA was synthesized using 500 ng of total RNA, 100 ng of *Escherichia coli* tRNA as a carrier^77^, specific primers, and an OT-1 kit for reverse transcription (Syntol) according to the manufacturer’s recommendations. Each RT reaction was performed in triplicate. Sequences of gene-specific primers and probes are presented below.

Ccn1-for 5′-CAAGGGGCTGGAATGCAATTTC

Ccn1-rev 5′-CCGTTCTGGTAGATCCTGGAGTTA

Ccn1-probe 5′-VIC- AGGGATCTGCAGAGCTCAGTCAGAAGGCA-BHQ2

Fos-F 5′- GGGACAGCCTTTCCTACTACC

Fos-R 5′- TGGCACTAGAGACGGACAGA

Fos-P 5-VIC- CCAGCCGACTCCTTCTCCAGCATG-BHQ2

Dusp1-F 5′- GCTGAGTACTAGTGTGCCTG

Dusp1-R 5′- GTCGAGCATATCTTTCCGGG

Dusp1-P 5′-VIC- CTTCCTGTACCTGGGCAGTGCTTA-BHQ2

Klf2-F 5′- CGGCAAGACCTACACCAAGAG

Klf2-R 5′- GCCGTCCCAGTTGCAATGATA

Klf2-P 5′-VIC- CTCACCTGTGTGCGTCCTCAGATGCG-BHQ2

Zfp36-F 5′- CGACTGTCGGTCTCTTCTCCA

Zfp36-R 5′- GACTCAGTTCCTCCGTGGTC

Zfp36-P 5′-VIC- TGACAGGTCATGGCTCATCGACATAAGGCTCTC-BHQ2

Psmd6-F 5′-CTGGGAGAAAGTGAAATTCGAGATG

Psmd6-R 5′-GGCCACAGTCTTATCATATGTCTTG

Psmd6-P 5′-VIC-CCTCCTTGTCACCTATCTGACAGAGGTACTCTGCTT-BHQ2

Sars-F 5′-ACCCAGCCCTCATTCGAGAG

Sars-R 5′-TCAGCTTGTTCAAGTTGTCTGC

Sars-P 5′-VIC-CGTCGCCACTCGCTGTCTGCCTTCAC-BHQ2qPCR was performed using cDNA, which was diluted 50 times in an aqueous solution of *E. coli* tRNA. One hundred nanograms of the carrier^77^, PCR reagents (Syntol), and a StepOnePlus System (Applied Biosystems) were then added to each reaction tube. Thermal cycling was performed as follows: (1) 600 s at 95 °C and (2) 45 cycles: 5 s at 95 °C followed by 10 s at 60 °C. qPCR was replicated three times for each cDNA. Psmd6 and Sars transcripts were used as references to normalize gene-expression data.

### Statistical analysis

The sequences of the primers and probes for expression analysis of the chosen genes *Ccn1*, *Dusp1*, *Fos*, *Klf2*, and *Zfp36* and reference genes *Psmd6* and *Sars* were designed using Beacon Designer 7.0 software (Premier Biosoft International).

Relative levels of the transcripts in the experimental groups were calculated as R = 2^(– ΔΔCt)^78^. The levels of the transcripts studied in the control group were set as 1. The significance of group differences was evaluated using the nonparametric Kruskall–Wallis test in the R programming language.

### RNA-seq data analysis

Ambiguous and low-quality bases were removed from FASTQ files using AdapterRemoval V2^79^.

Trimmed files were aligned to transcriptome obtained from an Rnor 6.0 assembly of rat genome and Rnor 6.0.93 gene annotation using the RSEM^80^ command rsem-prepare-reference with the –star option to also generate STAR indices^81^.

Alignment was performed using STAR and RSEM through the command rsem-calculate-expression with the –star option. Obtained pseudocounts were normalized using TMM algorithm normalization from the R “edgeR” package, command “calcNormFactors”^82^ and CPM algorithm from the R “limma” package, and “voom” command^83^. Mapped reads per sample and mapping percentages can be found in Supplementary File 4. PCA was conducted in order to identify potential outliers and batch effects. Plots of first four PCs can be found in Supplementary file 5.

A batch-effect correction was performed using the ComBat command from the R package “sva”^84^.

To detect differentially expressed genes, normalized reads were processed with the command (estimation of mean-dispersion ratios, determination of weights of observations), “lmFit” (creating a linear model describing the observations) and “eBayes” (determining the parameters of the aforementioned model) from the “limma” R package^83^.

Downstream analysis was performed on DEGs that were defined as genes in which the FC between control and experiment was higher than 1.5 and the *p* value of a moderated *t* test performed by limma with FDR multiple test correction was less than 0.05.

Raw and processed data can be accessed in the Gene Expression Omnibus, accession number GSE162524.

### GO term enrichment

Gene ontology term enrichment^85^ was performed using the ClueGO v. 2.5.3^86^ and Cluepedia v. 1.5.3^87^ apps for Cytoscape v. 3.6.1.

Terms were determined to be significantly enriched with the help of a hypergeometric enrichment test with correction of FDR (*p* < 0.05) with at least two genes related to the term. Terms were grouped by ClueGo based on the amount of common genes (> 50%).

### Gene networks

Gene-interaction networks were constructed using Pathway Studio® v. 12.1.0.9 (Elsevier, Netherlands).

Target genes and transcription-factor interaction networks were constructed using the R package “RTN”^88,89^, according to the developer’s recommendations. A description of the used code and commands can be found in Supplementary File 2.

## Conclusions

In our study, the transcriptome of hippocampus tissues derived from rats subjected to FST 20 min and 24 h after the test was examined. We identified significant changes in the transcription of 14 genes in the 20-min group, but not in the 24-h group, which demonstrated that the hippocampus transcriptome was affected by an immediate stress reaction after the FST, which subsides over the following 24 h.

A bioinformatics analysis of these genes revealed a group of interacting DEGs, namely, *Fos*, *Klf2*, *Ccn1*, *Zfp36*, and *Dusp1*. Based on this analysis, we believe that these genes play an important role in the immediate response to stress in the hippocampus. Four of these genes were previously found to be related to the immediate or delayed effects of stress exposure, which indirectly supports our findings.

It has also been shown that some of these five genes exhibit a significant change in their expression in the PFC 20 min (*Dusp1*, *Klf2*, *Zfp36*, and *Ccn1*) and 24 h (*Dusp1* and *Fos*) after the test. This suggests that the PFC is a brain area in which a delayed response to acute stress also occurs.

Of the five aforementioned genes, *Dusp1* is especially important, as its role in the pathogenesis of depression was demonstrated in both various animal stress models and in patients with depressive disorders. The significant differential expression in the brains of rats subjected to the FST indicates a possible commonality of molecular mechanisms acting in different rodent stress models.

## Study limitations

1. Within the scope of this study we haven’t conducted a measurement of relative changes of levels of proteins, encoded by genes mentioned in conclusion. Changes in the levels of mRNA don’t necessarily lead to changes in the levels of encoded proteins, which warrants caution when interpreting the applicability of findings of this study.
2. It is worth reiterating that FST is a very difficult model/assay to interpret. Despite its widespread use for antidepressant screening, FST’s relation to depression and interpretation of immobility of rats as “depressive phenotype” is dubious. We believe that it is worth mentioning that certain DEGs that we have identified have previously were found to be linked with depressive disorders and suicidality, but despite that we would like to state once again that within this work we interpret FST as a stressor, rather than a model of depression.
3. Measurements of immobility were not taken within this study, which makes it difficult to demonstrate the impact of FST on rats’ behavior.
4. Exclusively male rats were used in this study. As it was mentioned in discussion, changes of brain transcriptome after stress and/or depression both appear to be affected by sex. This suggests that data obtained in studies that focus on male rats exclusively is incomplete.

## Supplementary Information

Supplementary Information.

## Data Availability

The datasets generated and/or analysed during the current study are available in the GEO repository GSE162524.
