# Supplementary material for: Differential expression of Dusp1 and immediate early response genes in the hippocampus of rats, subjected to forced swim test
Source: Sci Rep. 2023 Jun 20;13:9985. doi: 10.1038/s41598-023-36611-5 (PMC10281975; doi:10.1038/s41598-023-36611-5)

Supplementary file 1 – Full table of DEGs with logarthimic fold change and p.values

Ensembl_gene_id	Gene_symbol	Gene_name	log_fc_limma	p.val	p.val.adjusted
ENSRNOG00000001189	Sik1	salt-inducible kinase 1 [Source:RGD Symbol;Acc:69407]	1.0643714667179	3.21745618883734e-06	0.00754761597631427
ENSRNOG00000003300	Btg2	BTG anti-proliferation factor 2 [Source:RGD Symbol;Acc:2225]	0.805995064632722	1.73573857696541e-05	0.0203587670589901
ENSRNOG00000003745	Atf3	activating transcription factor 3 [Source:RGD Symbol;Acc:2165]	1.42908271356901	7.01081563774836e-06	0.0109641366779231
ENSRNOG00000003977	Dusp1	dual specificity phosphatase 1 [Source:RGD Symbol;Acc:620897]	1.73378035536197	7.01041225124705e-09	4.93357762181511e-05
ENSRNOG00000007830	Apold1	apolipoprotein L domain containing 1 [Source:RGD Symbol;Acc:1303123]	2.4771259296382	6.39494047714083e-11	9.00087872157572e-07
ENSRNOG00000008015	Fos	Fos proto-oncogene, AP-1 transcription factor subunit [Source:RGD Symbol;Acc:2626]	1.26130470692187	5.06418617919547e-06	0.010062786812168
ENSRNOG00000014205	Klf2	Kruppel-like factor 2 [Source:RGD Symbol;Acc:1359220]	1.07173127854163	2.38695220880938e-05	0.0239973945278514
ENSRNOG00000014350	Cyr61	cysteine-rich, angiogenic inducer, 61 [Source:RGD Symbol;Acc:620763]	3.3357917912307	2.66727287876128e-08	0.000125139552561883
ENSRNOG00000016299	Klf4	Kruppel like factor 4 [Source:RGD Symbol;Acc:621445]	0.784834966729193	5.71952358773315e-06	0.010062786812168
ENSRNOG00000032585	AABR07048992.1		-3.59397359572471	2.9419726085918e-05	0.0276055096439531
ENSRNOG00000053462	RF00397	predicted gene, 26397 [Source:MGI Symbol;Acc:MGI:5456174]	-1.03970975411185	4.5962404176868e-05	0.034048465199443
ENSRNOG00000054344	Ier2	immediate early response 2 [Source:RGD Symbol;Acc:1359581]	1.01964460179399	1.83977627841372e-07	0.000517897022373461
ENSRNOG00000055160	AABR07033249.1		0.903000755697468	3.86043628330413e-05	0.033959775429691
ENSRNOG00000058388	Zfp36	zinc finger protein 36 [Source:RGD Symbol;Acc:620722]	1.11665725470846	6.70837896713499e-08	0.000236051084906062

Supplementary file 2 Code for running RTNI for R language

```
#These lines import required libraries
suppressMessages(library(RTN))
suppressMessages(library(biomaRt))
suppressMessages(library(limma))
suppressMessages(library(edgeR))

#This line imports rat mart to be used with getBM queries further
mart_gene_names<-
useDataset("rnorvegicus_gene_ensembl",mart=useMart("ENSEMBL_MART_ENSEMBL"))
#Path to the location of file with by sample raw counts
expression<-c("/media/ivan/DS1/data_storage/count_matrixes/fst_rsem_raw.txt")
#Load the data with by sample raw counts
expression_df<-as.matrix(read.table(expression))

#Create DGElist from counts matrix. Add 1 to every cell in the matrix to avoid error with log(0)
going forward.
dge <- DGEList(counts=(expression_df+1))
#Apply TMM normalization to DGElist
dge <- calcNormFactors(dge, method="TMM")

#Apply CPM normalization to DGElist
expr_logcpm_df<-log(cpm(dge),base=2)

#Get external gene names and descriptions from ensembl gene name. This is done in order to make
graph more readable on the output
expression_gene_names_query<-
getBM(values=rownames(expression_df),filters=("ensembl_gene_id"),attributes=c("ensembl_gene
_id","external_gene_name","description"),mart=mart_gene_names)
expression_gene_names_query[,2]<-make.unique(expression_gene_names_query[,2])

#Use unique gene names to link back later to gene descriptions and ensembl name.
rownames(expr_logcpm_df)<-expression_gene_names_query[,2]
rownames(expression_gene_names_query)<-expression_gene_names_query[,2]

colnames(expression_gene_names_query)<-c("ENSEMBLID","SYMBOL","Description")
#Path to and upload of a list of mammal transcription factors
input_tf_list<-c("/data/tf_data/rats_fst/tf_checkpoint.txt")
input_tf_df<-read.table(input_tf_list,sep="\t",header=TRUE,stringsAsFactors=FALSE)

#Path to and upload of list of DEGs or expression fold changes and p.values from statistical tests
```

```
input_degs<-c("/data/rats_models_of_depression/RSEM/all/fst_20m_combat_rsem_limma_result.c
sv")
input_degs_df<-read.table(input_degs,sep="\t",header=TRUE,quote="",stringsAsFactors=FALSE)

#Get external gene names, descriptions and Entrez gene ids for TF factors from TF factor lists
gene_names_query_1<-
getBM(values=input_tf_df[,1],filters=("external_gene_name"),attributes=c("ensembl_gene_id","ext
ernal_gene_name","description","entrezgene_id"),mart=mart_gene_names)

#Remove tf that are not present in rat genome
entrezids<-input_tf_df[(input_tf_df[,3]!="0"),3]

#Check if all TFs were found.
included<-tolower(input_tf_df[,1]) %in% tolower(gene_names_query_1[,2])
missing<-input_tf_df[!included,]
missing_aliases<-missing[,6]

#Perform additional search for the missing ones and combine it with the ones already found. This
step is optional
missing_aliases<-missing_aliases[(missing_aliases!="-") & (missing_aliases!="")]
splat<-unlist(strsplit(missing_aliases,"\\|"))
gene_names_query_2<-
getBM(values=splat,filters=("external_gene_name"),attributes=c("ensembl_gene_id","external_gen
e_name","description","entrezgene_id"),mart=mart_gene_names)
gene_names_query_full_output<-rbind(gene_names_query_1,gene_names_query_2)

#Filter out non degs. This step is also optional, if you have only uploaded degs
input_degs_only_df<-input_degs_df[(input_degs_df[,6] <= 0.05) &
(abs(input_degs_df[,4])>=log(1.5,base=2)),]

#Filter out TFs that are not differentially expressed
input_tfs<-input_degs_only_df[input_degs_only_df[,1] %in% gene_names_query_full_output[,1],]
#Add all zfp's from DEG lists
grepped_zfp<-grep("zfp",input_degs_only_df[,2],ignore.case = TRUE)
input_tfs<-rbind(input_tfs,input_degs_only_df[grepped_zfp,])


#Create tni object for further work
rtni <- tni.constructor(expData=expr_logcpm_df, regulatoryElements=as.character(input_tfs[,2]),
rowAnnotation=expression_gene_names_query[,c(2,1,3)])

#The tni.permutation function takes the pre-processed TNI object and returns a transcriptional
network inferred by mutual information (with multiple hypothesis testing corrections).
rtni <- tni.permutation(rtni, verbose = FALSE)

#In an additional step, unstable interactions can be removed by bootstrap analysis using the
tni.bootstrap function, which creates a consensus bootstrap network (referred here as refnet).
rtni <- tni.bootstrap(rtni)

#In the TN each target can be linked to multiple TFs and regulation can occur as a result of both
direct (TF-target) and indirect interactions (TF-TF-target).
#The Data Processing Inequality (DPI) algorithm (Meyer, Lafitte, and Bontempi 2008) is used to
```

```
remove the weakest interaction in any triangle of two TFs and a common target gene,
#thus preserving the dominant TF-target pairs, resulting in the filtered transcriptional network
(referred here as tnet).
#The filtered TN has less complexity and highlights the most significant interactions.
rtni <- tni.dpi.filter(rtni)

#All results available in the TNI object can be retrieved using the tni.get function:
tni.get(rtni, what="summary")
refnet <- tni.get(rtni, what="refnet")
tnet <- tni.get(rtni, what="tnet")

#The inferred transcriptional network can also be retrieved as an igraph object (Csardi and Nepusz
(2006)) using the tni.graph function.
#The graph object includes some basic network attributes pre-formatted for visualization in the R
package RedeR (Castro et al. 2012).

g <- tni.graph(rtni,minRegulonSize=2)
plot(g)
```

Supplementary file 3 – table with measurements taken in order to divide animals into groups. Time point one was upon animal arrival to the IMG, time point 2 was approximately one month later, after handling, approximately two weeks before the experiment. Animals that were sequenced are highlighted in color. The control group was highlighted using green color, the group, decapitated 24 hours after FST was highlighted using blue color, and the group, decapitated 20 minutes after FST was highlighted using red color. Animals, which didn't end up being sequenced are not highlighted.

Rat_number	Group	Weight_1_g	Food_consumption_1_g	Food_consumption_to_weight_ratio_1	Weight_2_g	Food_consumption_2_g	Food_consumption_to_weight_ratio_2	Sucrose_consumption_1_ml	Water_consumption_1_ml	Total_liquid_consumption_ml	Sucrose_preference_1	Sucrose_consumption_2_ml	Water_consumption_2_ml	Total_liquid_consumption_ml	Sucrose_preference_2
1	FST_20_m	139	20	0,144	186	22	0,118	51	2	53	0,962	46	2	48	0,958
2	FST_20_m	144	19	0,132	209	24	0,115	44	3	47	0,936	34	3	37	0,919
5	FST_20_m	118	15	0,127	161	18	0,112	20	2	22	0,909	21	4	25	0,840
6	FST_20_m	127	16	0,126	173	22	0,127	36	1	37	0,973	40	2	42	0,952
7	FST_20_m	126	15	0,119	178	21	0,118	79	2	81	0,975	61	2	63	0,968
10	FST_20_m	111	13	0,117	163	18	0,110	61	2	63	0,968	46	3	49	0,939
11	FST_20_m	117	17	0,145	167	17	0,102	72	8	80	0,900	17	28	45	0,378
12	FST_20_m	113	17	0,150	165	20	0,121	35	2	37	0,946	61	2	63	0,968
13	FST_24_h	115	19	0,165	161	18	0,112	45	3	48	0,938	47	1	48	0,979
14	FST_24_h	118	19	0,161	166	17	0,102	45	2	47	0,957	37	2	39	0,949
15	FST_24_h	133	17	0,128	186	19	0,102	44	15	59	0,746	87	2	89	0,978
16	FST_24_h	111	14	0,126	159	16	0,101	56	1	57	0,982	17	8	25	0,680
17	FST_24_h	118	14	0,119	174	17	0,098	44	1	45	0,978	31	1	32	0,969
18	FST_24_h	123	16	0,130	178	17	0,096	68	4	72	0,944	77	2	79	0,975
19	FST_24_h	118	22	0,186	182	23	0,126	45	2	47	0,957	33	3	36	0,917
20	FST_24_h	137	21	0,153	195	18	0,092	36	4	40	0,900	9	16	25	0,360
21	control	98	15	0,153	153	18	0,118	40	2	42	0,952	26	1	27	0,963
22	control	113	16	0,142	161	17	0,106	60	3	63	0,952	60	2	62	0,968
23	control	105	11	0,105	156	18	0,115	31	8	39	0,795	17	10	27	0,630
24	control	122	16	0,131	176	19	0,108	44	3	47	0,936	45	1	46	0,978
25	control	107	11	0,103	155	19	0,123	113	2	115	0,983	85	2	87	0,977
26	control	107	11	0,103	157	16	0,102	41	1	42	0,976	17	8	25	0,680
27	control	101	12	0,119	143	16	0,112	22	2	24	0,917	29	1	30	0,967
28	control	123	16	0,130	167	18	0,108	60	2	62	0,968	58	2	60	0,967

Supplementary file 4 – table with a number of reads after trimming, a number of aligned reads and a fraction of mapped reads per sample.

**Rat number**	**Group**	Trimmed reads	Aligned reads	Fraction mapped
1	FST_20_m	15440765	13130264	85,04%
2	FST_20_m	16511999	14123949	85,54%
5	FST_20_m	16160364	13212649	81,76%
7	FST_20_m	16173143	12610203	77,97%
10	FST_20_m	14283555	12528335	87,71%
11	FST_20_m	13822909	11635467	84,18%
12	FST_20_m	14283555	12528335	87,71%
14	FST_24_h	16323092	13364753	81,88%
15	FST_24_h	14890201	12618249	84,74%
16	FST_24_h	15697813	12902778	82,19%
17	FST_24_h	13198611	11776113	89,22%
18	FST_24_h	15860082	14123786	89,05%
19	FST_24_h	12594202	11083912	88,01%
22	control	18527114	15947947	86,08%
24	control	15284875	12780104	83,61%
25	control	16114127	13210342	81,98%
26	control	14933519	12918563	86,51%
27	control	12837432	11026411	85,89%
28	control	12042668	10784538	89,55%

Supplementary file 5 – plot of first 4 principal components from PCA. Analysis was conducted on CPM of counts per gene. PCs correspond to 22%,11%,9% and 6% (49% cumulatively) of total variance. Color denotes the batch, pch denotes the FST state.

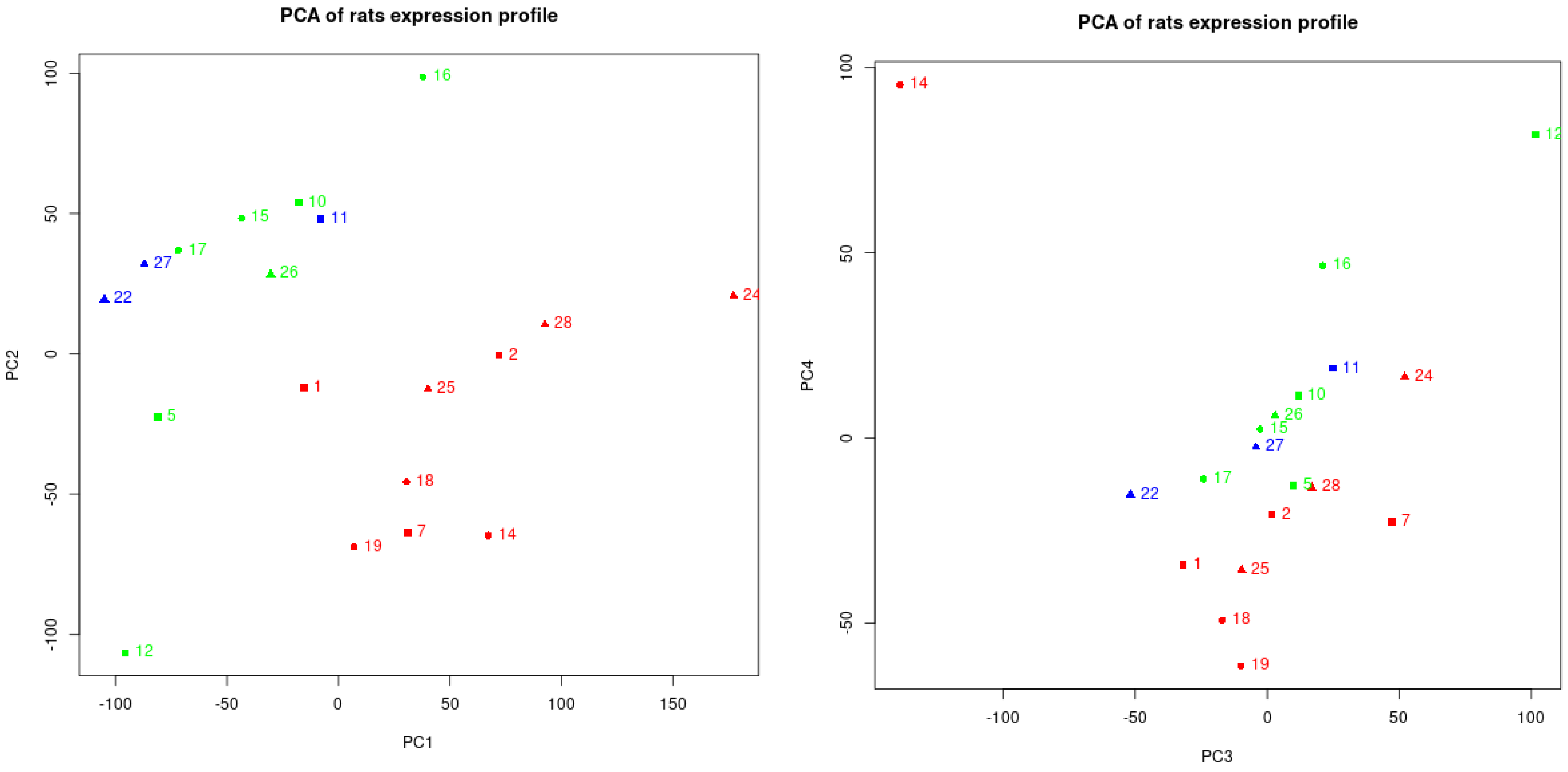

Supplement: Supplementary file 1 — Supplementary Information. [file 41598_2023_36611_MOESM1_ESM.pdf]
